# Imbalance in the ratio between mineralocorticoid and glucocorticoid receptors and neurodegeneration in the dentate gyrus of aged dogs

**DOI:** 10.14202/vetworld.2022.2543-2550

**Published:** 2022-11-10

**Authors:** Shirley Jaqueline Szriber, Leonardo Santana Novaes, Nilton Barreto Dos Santos, Carolina Demarchi Munhoz, Deise Carla Almeida Leite-Dellova

**Affiliations:** 1 Department of Veterinary Medicine, Faculty of Animal Science and Food Engineering, University of Sao Paulo, Duque de Caxias Norte Avenue, 225, 13635-900, Pirassununga, Brazil; 2 Department of Pharmacology, Institute of Biomedical Science, University of Sao Paulo, Professor Lineu Prestes Avenue, 1524, Room 323, 05508-000, Sao Paulo, Brazil

**Keywords:** aging, canine, cortisol receptors, hippocampus, neuronal loss

## Abstract

**Background and Aim:**

Cortisol binds to mineralocorticoid receptor (MR) and glucocorticoid receptor (GR) found in the hippocampus. The balanced expression of these receptors is essential to neuronal survival as MR and GR activations have antiapoptotic and proapoptotic effects, respectively. Given the aging changes in dogs’ dentate gyrus (DG) and the possible involvement of cortisol receptors in this process, this study aimed to evaluate the expression of MR and GR and neuronal degeneration in this hippocampal region of aged dogs.

**Materials and Methods:**

This study included cadaveric histologic hippocampus sections from six dogs aged 10 years and older (AG group) and 12 young/adult dogs aged up to 8 years (YAd group). Nissl staining and immunohistochemistry were performed to identify cells and investigate MR and GR expression, respectively. Furthermore, fluorescent labeling (fluoro-Jade B) was used to detect degenerating neurons.

**Results:**

The AG group’s polymorphic layer of the DG had a lower cell count (16%) and more degenerating neurons than the YAd group. In addition to these cellular changes, the AG group had lower MR immunoreactivity and MR-to-GR ratio. Furthermore, the lowest MR expression was associated with neuronal degeneration in the polymorphic layer of the DG of dogs.

**Conclusion:**

An imbalance in the MR-to-GR ratio was observed in the polymorphic layer of the DG of aged dogs, along with lower MR expression and a greater number of degenerating neurons. These findings have clinical implications for understanding the decline in hippocampal memory formation associated with cognitive changes in aged dogs.

## Introduction

The mineralocorticoid receptor (MR) and glucocorticoid receptor (GR) are highly expressed in the hippocampus, where they contribute to cognitive processes, such as information retrieval (through the MR pathway) and memory storage (through the GR pathway), acting in a complementary way during the stress response [1, 2]. Binding to glucocorticoids (GCs), such as cortisol and corticosterone, can activate MR and GR [2, 3]. The hormonal effects on the hippocampus are determined by the GC concentration, the GC binding affinity to MR and GR, the MR and GR expression levels, and the MR-to-GR ratio in nerve cells [4]. Indeed, higher GC concentrations activate GR, whereas basal GC concentrations occupy MR because GC affinity for MR is ten-fold greater than GR [2, 3, 5, 6]. *In vitro* studies have shown that the high levels of GR activation, as well as high cortisol concentrations, can be harmful to the hippocampus, whereas MR activation can have some neuroprotective effects [7, 8]. Crochemore *et al*. [7] found that the GR agonist dexamethasone promoted hippocampal neuronal death in rat hippocampal cells, which was reversed by the MR agonist aldosterone. High cortisol concentrations inhibit proliferation and neuronal differentiation in a human fetal hippocampal progenitor cell line through a GR-dependent mechanism, whereas low cortisol concentrations increase cell proliferation through an MR-dependent mechanism [8]. A correlation between brain atrophy and age-dependent cognitive decline in humans and dogs has been demonstrated [9]. Cortisol levels increased in older people [10], possibly due to hypothalamic-pituitary-adrenal axis feedback deregulation, are associated with cognitive deficits and hippocampalatrophy [11, 12]. Aged dogs may have higher plasmatic cortisol concentrations [13], which may contribute to hippocampus atrophy, loss, and cognitive impairment [14, 15]. However, this study focused on the expression of MR and GR and neurodegeneration in aged dogs rather than on circulating cortisol levels.

The dentate gyrus (DG) is a hippocampus subregion composed of molecular, granule cell, and polymorphic layers. The molecular layer is mainly occupied by dendrites from neurons in the other two DG layers and fibers from other cerebral regions, including the entorhinal cortex. The granule cell layer primarily consists of densely packed granule cells that project their axons to the polymorphic layer and the hippocampus’ cornu ammonis 3 (CA3) field. The mossy cells and hilar perforant path-associated cells in the polymorphic layer have excitatory and inhibitory effects on granule cells, respectively [16]. The DG plays a key role in hippocampal memory formation, with functions for nonspatial [17, 18] and spatial memory [17, 19, 20] and novelty detection [21]. Aging affects the memory system in humans and rodents, dependent on the connections between the DG and CA3 in the hippocampus [22]. Functional imaging studies on different hippocampal regions of aged rats and monkeys indicated that the DG is the hippocampal subregion most affected by aging [23]. Furthermore, neurons in the DG’s hilar region are lost in aged humans, rats, and dogs. Indeed, aged dogs had 30% fewer hilar neurons than younger dogs [15].

The aging of a dog’s central nervous system (CNS) is associated with cognitive decline and neuropathological changes, similar to humans [9, 14, 24, 25]. Given the increased canine life expectancy and the neurodegenerative processes associated with aging [24, 26], such as neuronal loss [14], it is critical to study the neuropathological processes in aged dogs in different breeds that are part of the clinical routine. In this way, it would be possible to understand under what conditions the CNS of this species is subjected to aging.

Considering the protective effect of MR [7], neuronal loss in the hippocampus [14], and susceptibility of the DG to aging [15, 22, 23], this study aimed to evaluate the expression of MR and GR in the DG of young/adult and aged dogs using postmortem tissue and immunohistochemistry technique. Furthermore, we evaluated the labeling for degenerating neurons to investigate the relationship between the expression of MR and GR and the degeneration of DG neurons in dogs.

## Materials and Methods

### Ethical approval

The experimental protocols were approved by the institution’s Ethics Committee on the Use of Animals (protocol number 2784290317).

### Study period and location

The study was conducted from January 2017 to January 2018. Samples of the canine hippocampus were collected at the Department of Veterinary Pathology, Faculty of Veterinary Medicine and Animal Science, University of Sao Paulo, Sao Paulo, Brazil. Hippocampus samples were processed at the Department of Pharmacology, Institute of Biomedical Science, University of Sao Paulo, Sao Paulo, Brazil.

### Hippocampus sample

Eighteen canine hippocampus samples were collected routinely at the Faculty of Veterinary Medicine and Animal Science, Department of Veterinary Pathology, University of Sao Paulo. Necropsies were performed within 12 h after the animal’s death. The necropsy record was used to collect the dog’s information (sex, age, body weight, breed, and presumptive diagnosis), as shown in Tables-1 and 2. Hippocampal samples were divided into two experimental groups: aged (AG; six aged dogs aged 10 years and older) and young/adult (YAd; 12 dogs aged up to 8 years). This study excluded dog samples that died from hepatic encephalopathy, diabetic ketoacidosis, or infectious disorders, such as distemper, parvovirus, toxoplasmosis, bacterial encephalitis, or head trauma.

**Table-1:** Relevant clinical data of young and adult dogs included in this study.

Dogs	Breed	Sex	Age	Weight (kg)	Presumptive diagnosis
1	Shih Tzu	M	3 m	2.0	Gastroenteritis
2	Mixed breed	M	6 m	4.9	Megaesophagus
3	Bull terrier	M	10 m	17.1	Congenital kidney disease
4	Mixed breed	F	3 y	13.2	Cardiorespiratory disease
5	Poodle	F	8 y	1.8	Acquired kidney disease
6	Dalmatian	F	8 y	ni	Gastroenteritis

M=male, F=female, m=month, y=year, ni=not informed

**Table-2:** Relevant clinical data of aged dogs included in this study.

Dogs	Breed	Sex	Age (years)	Weight (kg)	Presumptive diagnosis
1	Brazilian mastiff	M	10	26.5	Unknown/abdominal neoplasm
2	Labrador retriever	M	10	15.0	Gastroenteritis
3	Beagle	M	12	12.7	Cardiorespiratory disease
4	German shepherd	M	13	39.5	Cardiomyopathy
5	Yorkshire	M	14	6.8	Pancreatitis
6	Poodle	M	20	12.5	Unknown/senescence
7	Golden retriever	F	10	27.9	Osteoarticular disease
8	Poodle	F	11	6.2	Urinary tract disease
9	Mixed breed	F	12	11.2	Biliary obstruction
10	Mixed breed	F	14	9.9	Cardiorespiratory disease
11	Whippet	F	16	ni	Lymphoma
12	Mixed breed	F	26	ni	Chronic kidney disease

M=Male, F=Female, NI=Not informed

### Tissue preparation

Hippocampal samples were cryoprotected in 30% sucrose solution in phosphate-buffered saline (PBS, pH 7.4) at 4 C after being fixed in 10% formaldehyde solution for 24–72 h. They were immersed in Tissue-Tek optimal cutting temperature compound (Sakura Finetek, Torrance, CA, USA) for 48–72 h before being sliced in 40-μm-thick histological sections using a semiautomatic cryostat (Leica, model 1850 UV, Wetzlar, Germany) and stored at −20°C in an antifreeze solution (0.4 mol/L sodium phosphate buffer, 15% sucrose, and 30% ethylene glycol diluted in distilled water) until the histochemical procedures.

### Nissl staining for cell identification

The sections were mounted on gelatin-coated slides and immersed for 24 h in 70% ethanol. Then, the slides were dehydrated by successive immersion in 95% ethanol and hydrated in 98.5% xylol, followed by an ethanol gradient of 100%, 95%, 70%, and 50%. The slides were then rinsed with distilled water, incubated for 25 s in 0.25% thionine solution, and washed again. Next, another dehydration process was performed using sequential immersion in a gradient of 50%, 70%, and 95% ethanol, 95% ethanol plus 1% acetic acid, 95% ethanol, 100% ethanol, and 98.5% xylene. Finally, the slides were coverslipped with dibutyl phthalate polystyrene xylene (DPX) (Sigma-Aldrich, St. Louis, MO, USA) mounting media.

### Immunohistochemistry for MR and GR

Mineralocorticoid receptor and GR immunohistochemistry was performed in DG’s free-floating sections. After washing the sections with 0.02 mol/L potassium PBS (KPBS) to remove the antifreeze solution, they were incubated in 0.3% H_2_O_2_ to inactivate endogenous peroxidase. Following a few more washes, the sections were incubated in 0.01 mol/L citrate buffer at 60°C before being rinsed in 0.02 mol/L KPBS. The sections were then incubated for 40 min at room temperature (21 ± 2°C) in block solution (1% donkey serum and 0.3% Triton diluted in 0.02 mol/L KPBS). The sections were treated overnight at 4°C in a block solution containing antibodies goat polyclonal anti-MR (1:500, sc-6860) and rabbit polyclonal anti-GR (1:1000, sc-1004) (Santa Cruz Biotechnology, Dallas, TX, USA). The sections were then rinsed with 0.02 mol/L KPBS and incubated for 2 h at room temperature (21 ± 2°C) with the secondary antibodies (anti-goat IgG and anti-rabbit IgG [1:1000]) (Santa Cruz Biotechnology) in 0.02 mol/L KPBS and 0.3% Triton solution. Thereafter, the sections were incubated in the avidin–biotin complex (Elite kit; Vector, Burlingame, CA, USA) to visualize the antigen-antibody complex and tissue development with nickel, chromogen 3,3′-diaminobenzidine, and H_2_O_2_. After assembly and drying (overnight), the slides were exposed to a dehydration battery with sequential immersion in distilled water, followed by a gradient of 50%, 70%, 95%, and 100% ethanol and 98.5% xylol. Finally, the slides were coverslipped with DPX (Sigma-Aldrich) mounting media.

### Identification of degenerating cells with fluoro-Jade B

The sections were mounted on gelatin-coated slides and immersed in the following solutions: 80% ethanol plus 1% NaOH for 5 min, 70% ethanol for 2 min, ultrapure water for 2 min, 0.06% potassium permanganate for 10 min (protected from light), ultrapure water for 2 min, fluorine B (Millipore, Burlington, VT, USA) + 4′,6-diamidino-2-phenylindole for 20 min, and ultrapure water for 1 min (3 times). After drying, the slides were immersed in 98.5% xylol. Finally, the slides were coverslipped with DPX (Sigma-Aldrich) mounting media.

### Quantification of data and statistical analysis

**Supplementary Figure-1:**
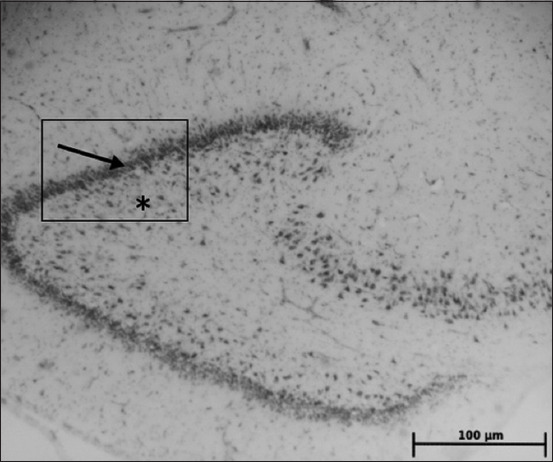
Established gyrus dentate region used for all the analyses. The granular (arrow) and polymorphic (*) layers of dog’s gyrus dentate are indicated (Nissl staining, 4×).

The analyses were performed by the same individual blinded to the DG sample origin (YAd group or AG group). Images obtained by the Digital Sight camera coupled to the Eclipse E600 (20×) optical microscope with NIS-Elements Advanced Research 2.30 Image Software (Nikon Instruments Inc., Tokyo, Japan) were used to determine cellular identification in the granule cell and polymorphic layers of DG and MR and GR immunoreactivity in the polymorphic layer of DG. Images produced by the Digital DXM 1200C camera attached to the E1000 (20×) optical microscope and viewed using a Control Software ACT-1 Imaging System (Nikon Instruments Inc.) were used to calculate the number of fluoro-Jade B positive neurons in the polymorphic layer of the DG. Six representative slices of the hippocampus were evaluated for each sample, and the average of these six slices corresponded to the result per sample. Supplementary Figure-1 shows the established DG region in each slice used for all analyses and the granule cell and polymorphic layers.

The analyses were performed using ImageJ software (National Institutes of Health, Bethesda, MD, USA). Nissl cellular identification in the granule cell layer was performed by cell density analysis in five consecutive fields (1.85 × 105 pixels^2^). Due to the overlap of neurons and glial cells in the granule cell layer, the analysis of GR, MR, and fluoro-Jade B labeling was limited to the polymorphic layer of DG.

The neurons positive for Nissl, MR, GR, and fluoro-Jade B staining in the polymorphic layer was counted in two consecutive fields (1.02 × 104 pixels^2^). The MR and GR were determined separately, and the MR-to-GR ratio was derived from these values. The percentage (%) of positive B-fluoro-neuronal neurons was calculated using the following formula: % = (counts of fluoro-Jade B neurons positive × 100)/cell count.

The result of each sample corresponded to the average of the evaluated fields and slices. For each group, the quantitative parameters are presented as the mean values with their respective standard errors of the mean. The results were subjected to analysis of variance by the *F* test, and the unpaired t-test performed comparisons between the YAd and AG groups. Correlation tests were applied between different parameters using the Pearson correlation coefficient. p < 0.05 and <0.1 indicated significant differences and tendency, respectively.

## Results

### Identification and quantification of cells in the granule cell and polymorphic layers of DG

**Figure-1:**
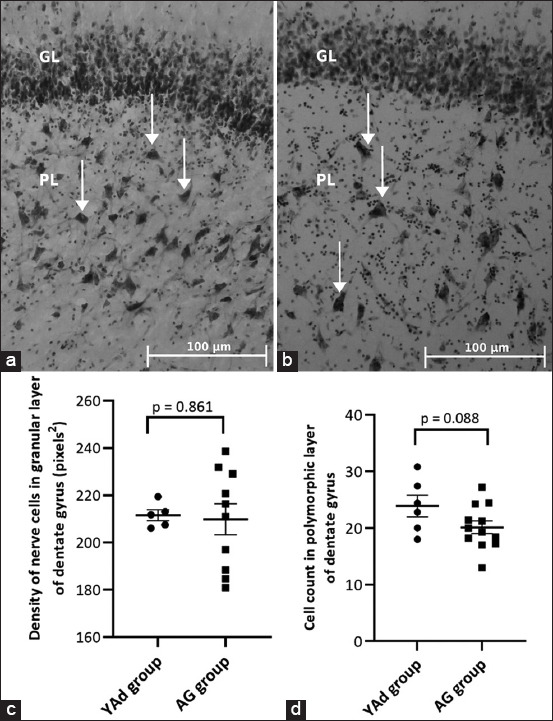
Microscopic view of the dentate gyrus of dogs of YAd and AG groups showing that in the polymorphic layer (PL) it is possible to individualize the cells (arrows) that seem to be more present in the sample of the young dog compared to the aged dog. (a) Shih Tzu, 3 months, male and (b) Whippet, 16 years old, female (Nissl staining, 20×). (c) Individual and mean (±SEM) values of density of cells in the granular layer. (d) Individual and mean (±SEM) values of cells count (number of cells) in the PL. There was no difference between groups (N: 5–12). SEM=Standard error of the mean.

A representative image of Nissl staining of the granule cell and polymorphic layers of dogs from the YAd and AG groups is presented in Figures-1a and b, respectively. There was no difference in the cellular density of the granule cell layer of the DG between the YAd and AG groups (211.6 ± 2.352 pixels^2^ vs. 209.9 ± 6.613 pixels^2^, respectively; p = 0.8613, Figure-1c). There was a tendency for a reduction in the number of cells in the polymorphic layer in the AG group compared with the YAd group (23.9 ± 1.926 vs. 20.1 ± 1.117, respectively; p = 0.0883, Figure-1d).

### Immunohistochemistry for MR and GR in cells of the polymorphic layer of the DG

**Figure-2:**
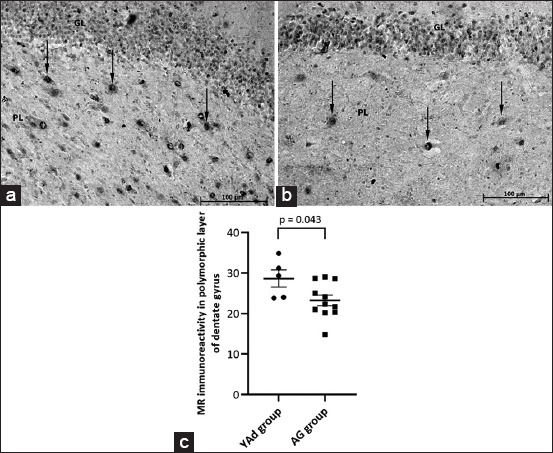
Microscopic view of the dentate gyrus of dogs of YAd and AG groups showing that abundant mineralocorticoid receptor (MR) immunoreactivity is present in the polymorphic layer of the adult dog (arrows) while the aged dog presents a few MR staining. (a) Poodle, 8 years old, female. (b) Beagle, 12 years old, male (20×). (c) Individual and mean (±SEM) values of MR immunoreactivity (number of cells). MR immunoreactivity was lower in the AG group (N: 5–11). SEM=Standard error of the mean.

**Figure-3:**
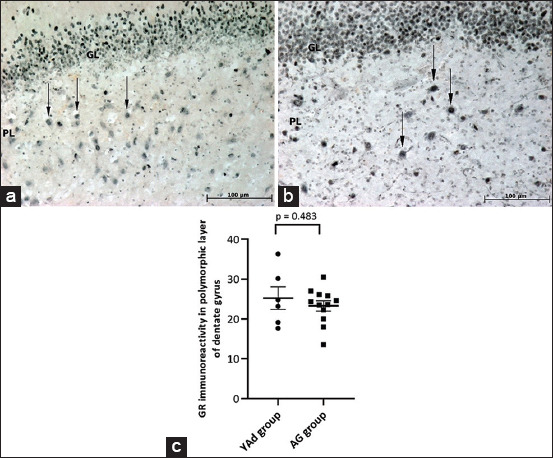
Microscopic view of the dentate gyrus of dogs of YAd and AG groups showing that abundant glucocorticoid receptor (GR) immunoreactivity (arrows) is present in the polymorphic layer of both dogs. (a) Shih Tzu, 3 months, male. (b) Golden, 10 years old, female (20×). (c) Individual and mean (±SEM) values of GR immunoreactivity (number of cells). There was no difference in the GR immunoreactivity between the groups (N: 6–12). SEM=Standard error of the mean.

Representative images of MR and GR immunoreactivity in the DG of dogs from the YAd and AG groups are presented in Figures-2a, b, and 3a, b, respectively. In the polymorphic layer, the AG group had fewer MR immunopositive cells than the YAd group (23.3 ± 1.322 vs. 28.6 ± 2.120, respectively; p = 0.0433, Figure-2c), whereas there was no difference in GR immunoreactivity between the YAd and AG groups (25.2 ± 2.864 vs. 23.3 ± 1.288, respectively; p = 0.4827, Figure-3c). These findings resulted in a lower MR-to-GR ratio in the AG group than in the YAd group (0.97 ± 0.04 vs. 1.31 ± 0.20; p = 0.0297).

### Identification and quantification of degenerating neurons in the polymorphic layer of the DG

**Figure-4:**
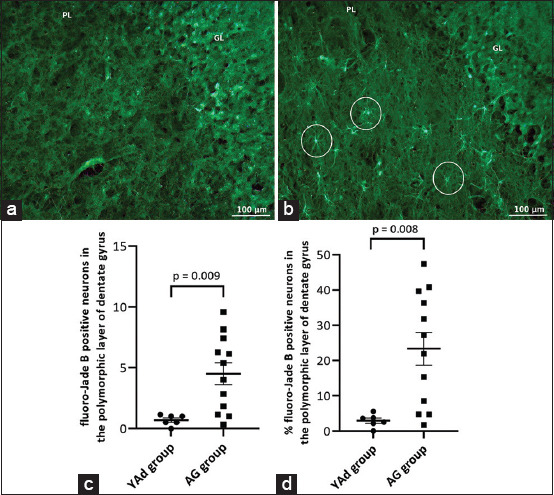
Microscopic view of the dentate gyrus showing fluoro-jade B positive neurons (circles) only in the aged dog. (a) Mixed breed, 6 months, male. (b) Beagle, 12 years old, male (20×). (c) Individual and mean (±SEM) of fluoro-Jade B positive neurons in the polymorphic layer (PL). (d) Individual and mean (±SEM) values of percentage (%) of fluoro-Jade positive neurons in the PL, in relation to the total number of cells. The number and % of fluoro-Jade positive neurons were higher in the AG group. N: 6–12. SEM=Standard error of the mean.

A representative image of fluoro-Jade B staining of granule cell and polymorphic layers of dogs from the YAd and AG groups is presented in Figures-4a and b, respectively. The total number of degenerating neurons was higher in the AG group than in the YAd group (4.5 ± 0.8936 vs. 0.69 ± 0.1790, respectively; p = 0.0092, Figure-4c). Similarly, the percentage of degenerating neurons was higher in the AG group than in the YAd group (2.95 ± 0.7628 vs. 23.4 ± 4.643, respectively; p = 0.0076, Figure-4d).

### Correlation analysis

We found a negative correlation between dog age and the number of cells in the polymorphic layer (*r* = −0.618, p = 0.038). There was a negative correlation between the number of fluoro-Jade B positive neurons and MR-positive cell immunoreactivity (*r* = −0.830, p = 0.030) and between the number of fluoro-Jade B positive neurons and the MR-to-GR ratio (*r* = −0.712, p = 0.016) in the polymorphic layer.

## Discussion

The hippocampus is divided into subregions with distinct synaptic pathways [27]. The DG receives multiple sensory (vestibular, olfactory, visual, auditory, and somatic) and spatial stimuli from the entorhinal cortex; therefore, lesions or degenerative processes in the DG may impair the processing of spatial (location) and nonspatial information (object and odor discrimination) [17].

According to Chapagain *et al*. [28], dogs with cognitive aging may exhibit decreased ability to find dropped food, erratic locomotion patterns, and a decline in learning and memory. Accordingly, a study reported that aging reduces discrimination learning ability in pet dogs [29], and these signs could indicate DG impairment [19]. Therefore, the DG was chosen as the subject of this study due to the relevance of this area. Furthermore, other authors have identified significant alterations in this region associated with canine brain aging, such as decreasing neuron counts and neurogenesis [15].

**Supplementary Figure-2:**
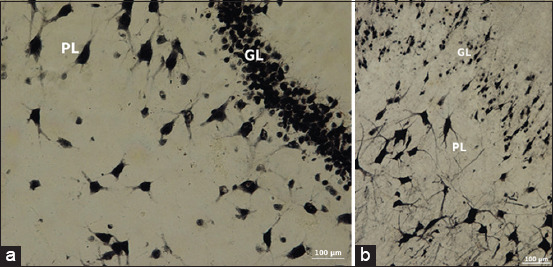
Neurons in the polymorphic and granular layer of the dentate gyrus of young/adult and aged dogs by NeuN. (a) Mixed breed, 6 months, male and (b) mixed breed, 26 years old, female. GL=Granular layer, PL=Polymorphic layer (20×).

The Nissl technique was chosen to perform the neuron identification because it stains the nerve cells differently, allowing the distinction of neurons [30]. Neuron identification was also confirmed by comparing the sizes and morphologies of neurons stained with NeuN in some samples (Supplementary Figure-2).

In this study, it was impossible to estimate the Nissl-positive cell count in the DG granular cell layer due to the overlapping of cells in the histological sections. Thus, a cell density analysis was performed in this layer, and no difference was observed between the YAd and AG groups. In the previous studies, researchers could count neurons in dogs’ DG granular cell layer using the NeuN immunohistochemical stain (a neuron-specific soluble nuclear antigen) [15, 20]. However, both cell density analyses using Nissl staining or NeuN-positive cell count indicate no significant reduction in the number of cells in the granular layer when young and old dogs are compared. Siwak-Tapp *et al*. [15] evaluated the DG of young (<5 years old) and aged (>13 years old) dogs and found that the number of neurons in the granule cell layer did not differ between groups. Hwang *et al*. [20] found no differences in the number of neurons in dogs up to 8 years of age but observed a slight reduction in NeuN-positive cells in three 12-year-old dogs. The AG group had the lowest cell density values (4/10) in dogs older than 10 years (Figure-1c); nonetheless, the AG group’s mean cell density value of the granular layer was not lower than the YAd group. Furthermore, there was no significant relationship between the dog’s age and the cell density of the DG’s granular layer.

Similarly, we employed NeuN staining to evaluate neurons’ morphology and size. However, contrary to what has been described in the literature, individualizing the cells in our samples was difficult (Supplementary Figure-2).

Individualization of histochemical markers (immunohistochemical and fluoro-Jade B) was further hampered by densely compacted cells in the DG granule cell layer, compromising the evaluation of receptors (MR and GR) and degenerating neurons. There were fewer cells in the polymorphic layer, and it was feasible to individualize the neurons; therefore, the histochemical analysis performed in this layer was presented.

In the AG group, there was a tendency for a cell count reduction in the polymorphic layer. We suggest that these cells are primarily neurons because of the similarities in size and morphology between the counted Nissl-stained cells and the NeuN-stained neurons in our analysis. Similarly, more fluoro-Jade B positive neurons in the AG group than in the YAd group suggested that cell decrease in the DG polymorphic layer includes lost neurons. The decrease in cell count is consistent with the literature because neurons in the hilar region (which includes the polymorphic layer) can be depleted with aging [22]. Furthermore, Siwak-Tapp *et al*. [15] demonstrated that in canines, this reduction could be up to 30%. This study corresponded to 16%, and we identified a negative correlation between the dogs’ ages and the number of cells counted in the polymorphic layer.

The AG group had decreased MR expression compared with the YAd group, whereas the GR labeling remained the same. Choi *et al*. [13] observed a reduction in immunoreactivity for both receptors in the DG of aged dogs (aged 10–12 years). However, with higher cortisol levels in the serum of aged dogs, the reduction in MR immunoreactivity was much more expressive. Reul *et al*. [31] also showed a reduction in MR but not GR levels in the hippocampus of aged dogs (over 11 years old) by studying the MR and GR binding to their particular ligands. In contrast to these findings, Murphy *et al*. [32] found no alteration in the MR and GR expression levels in the DG of the hippocampus of aged rats.

The fluoro-Jade B staining was used to selectively detect neuron death [33]. The AG group presented a higher number of degenerating neurons (positive fluoro-Jade B staining) in the polymorphic layer, decreased MR expression, and a tendency to have fewer cells in this region. Neuronal loss in the dog’s brain is present in the elderly [14, 15, 24], and it is a possible cause of atrophy of the hippocampus and other CNS regions found in aged dogs [14]. Accordingly, researchers have identified age-related changes in hippocampal anatomy, such as reducing the height of the hippocampal formation in aged dogs using magnetic resonance imaging [34].

Previous studies have suggested that MR activation has a significant protective effect, preventing the GR-mediated apoptosis of hippocampal neurons [7, 35]. However, GR alone did not appear to play a significant role in the cellular and structural alterations of the DG, as there was no difference in GR labeling between the YAd and AG groups. Nonetheless, the AG group’s tendency toward decreased cell quantity and higher numbers of degenerating neurons may be related to the AG group’s lower MR expression and MR-to-GR ratio. Indeed, there was a negative correlation between the number of degenerating neurons and MR labeling and the MR-to-GR ratio in the DG polymorphic layer.

The neuroprotective effects of MR occur particularly in DG cells, and MR overexpression appears to protect the DG from the effects of excess GC on nonspatial memory in rats [18]. Adrenal steroids regulate DG neurogenesis [36]. Woolley *et al*. [37] demonstrated that adrenalectomized rats showed increased apoptosis in DG cells, which was completely reversed by aldosterone treatment due to MR agonism because aldosterone is a mineralocorticoid hormone that binds to MR several orders of magnitude higher than to GR [38]. Thus, one can observe the significance of the MR and balanced MR-to-GR ratio in the function and survival of DG neurons.

Our findings are consistent with those of Choi *et al*. [13], who observed a reduction in MR immunoreactivity in postmortem hippocampus samples of aged dogs. However, in this study, only male brain samples were used, and there was neither association between the imbalance of MR-to-GR ratio (due to low MR expression) and neuronal degeneration present in canine cerebral aging, nor between hippocampus samples from dogs of different breeds and ordinary clinical practice. Thus, our findings are important for evaluating MR and GR expression and neuronal integrity in the DG of aged dogs.

### Limitations of the study

There were some limitations of this study. Due to the overlapping of neuronal cells in the granular cell layer, the analysis of GR, MR, and fluoro-Jade B labeling was limited to the polymorphic layer of the DG. There was no information available on the dog’s behavior and cortisol levels, which would aid in understanding the impact of reduced MR expression and a greater number of degenerating neurons in the polymorphic layer of the DG of aged dogs. A larger experimental N, particularly in the AG group, could allow comparisons of male and female dogs and dogs of various sizes. However, we believe that the experimental N was sufficient to support the article’s conclusions.

## Conclusion

The results of this study suggest that an imbalance in the MR-to-GR ratio, with reduced MR expression, may be related to neurodegeneration in the DG of aged dogs. Clinically, the relevance of these findings is that the increased rate of degenerating neurons found in the DG could contribute to the decline in hippocampal memory formation and partly explain the cognitive alterations observed in aged dogs. Future research will have to determine how cortisol affects the MR and GR expressed in the DG of aged dogs and whether a differentiated activation of the MR over the GR could reduce the number of degenerated neurons in the polymorphic layer of DG.

## Authors’ Contributions

CDM, SJS, LSN, and NBDS: Conceptualized and designed the study. SJS: Collected the samples and performed the experiments. SJS and DCAL: Analyzed and interpreted the data and wrote the manuscript. All authors have read and approved the final manuscript.
